# Exhaled Breath Analysis for Diabetes Diagnosis and Monitoring: Relevance, Challenges and Possibilities

**DOI:** 10.3390/bios11120476

**Published:** 2021-11-25

**Authors:** Kaushiki Dixit, Somayeh Fardindoost, Adithya Ravishankara, Nishat Tasnim, Mina Hoorfar

**Affiliations:** 1 Department of Electronics and Electrical Communication Engineering, Indian Institute of Technology Kharagpur, Kharagpur 721302, India; kdixit00@iitkgp.ac.in; 2 School of Engineering, University of British Columbia, Kelowna, BC V1V 1V7, Canada; Fardindoost@physics.Sharif.edu (S.F.); arka1995@mail.ubc.ca (A.R.); nishat.tasnim@ubc.ca (N.T.); 3 Faculty of Engineering and Computer Science, University of Victoria, Victoria, BC V8W 2Y2, Canada

**Keywords:** diabetes, non-invasive detection, exhaled breath analysis, breath sensor, volatile organic compounds, blood glucose monitoring, biomarkers

## Abstract

With the global population prevalence of diabetes surpassing 463 million cases in 2019 and diabetes leading to millions of deaths each year, there is a critical need for feasible, rapid, and non-invasive methodologies for continuous blood glucose monitoring in contrast to the current procedures that are either invasive, complicated, or expensive. Breath analysis is a viable methodology for non-invasive diabetes management owing to its potential for multiple disease diagnoses, the nominal requirement of sample processing, and immense sample accessibility; however, the development of functional commercial sensors is challenging due to the low concentration of volatile organic compounds (VOCs) present in exhaled breath and the confounding factors influencing the exhaled breath profile. Given the complexity of the topic and the skyrocketing spread of diabetes, a multifarious review of exhaled breath analysis for diabetes monitoring is essential to track the technological progress in the field and comprehend the obstacles in developing a breath analysis-based diabetes management system. In this review, we consolidate the relevance of exhaled breath analysis through a critical assessment of current technologies and recent advancements in sensing methods to address the shortcomings associated with blood glucose monitoring. We provide a detailed assessment of the intricacies involved in the development of non-invasive diabetes monitoring devices. In addition, we spotlight the need to consider breath biomarker clusters as opposed to standalone biomarkers for the clinical applicability of exhaled breath monitoring. We present potential VOC clusters suitable for diabetes management and highlight the recent buildout of breath sensing methodologies, focusing on novel sensing materials and transduction mechanisms. Finally, we portray a multifaceted comparison of exhaled breath analysis for diabetes monitoring and highlight remaining challenges on the path to realizing breath analysis as a non-invasive healthcare approach.

## 1. Introduction

Diabetes mellitus (DM) is a severe chronic metabolic disease that affects around 463 million people globally [1]. An estimated 4.2 million deaths among adults in 2019 were attributed to DM, which is equivalent to one death every 8 s [1]. DM can be broadly categorized as type 1 (T1DM), type 2 (T2DM), and gestational. T1DM is caused by β-cell destruction in the pancreas, leading to absolute insulin deficiency [2]. T2DM is an outcome of insulin resistance and is associated with comorbidities such as obesity, hypertension, and dyslipidemia [2]. Gestational Diabetes is the onset of diabetes during pregnancy due to increased adiposity and hormonal variations caused by the placenta, resulting in insulin resistance [3]. Additionally, there are other forms of diabetes, such as monogenic diabetes syndromes, exocrine pancreas diseases, and drug-induced diabetes [2]. Diabetes has inevitable detrimental effects on the quality of life, including issues with psychological and physiological functioning, risk of developing comorbidities, and the financial burden of insulin treatment. Despite being crucial for T1DM patients, insulin is not readily globally accessible due to affordability and availability issues. A survey of 15 countries found only four countries with 100% insulin availability and six with greater than 80% availability [4]. The median cost of human insulin and analogue insulin was around USD 7.64 and USD 5.90, respectively, in the public sector. Comparatively, these prices in the private sector were 2.8 and 5.2 times higher per 10 mL 100 IU vial equivalent [1]. People with diabetes are prone to developing cardiovascular diseases, eye disorders, neuropathy, and nephropathy. There is a 20% higher risk of breast cancer and a two-fold greater risk of developing endometrial and intrahepatic cholangiocarcinoma among adults with T2DM and a high body mass index (BMI) [1]. These acute and long-term complications create enormous burdens on the healthcare economy. Direct costs comprise providing preventative and curative health services, family planning activities, nutrition activities, and emergency aid. In contrast, premature deaths, workplace absenteeism and presenteeism, and loss of labour due to disabilities contribute to indirect costs [1]. The annual global health expenditure on diabetes is expected to reach USD 845 billion by 2045 [1].

Despite the severity of the disease, the state of its global screening is unsatisfactory. Approximately 50.1% of adults with diabetes are unaware of their health condition [1]. This justifies the need for a simple diabetes monitoring system that can be employed for community screening. Regular blood glucose monitoring is an effective strategy for personalized diabetes management, as blood glucose fluctuations with different activities or illnesses need to be considered to plan an appropriate lifestyle. Currently, there are various invasive, minimally invasive, and non-invasive devices available in the market for blood glucose monitoring. Invasive self-monitoring blood glucose devices require finger-pricking, up to even 10 times per day [5]; however, the associated pain and risk of infection make these devices inconvenient. Minimally invasive devices generally target interstitial fluid, requiring subcutaneous sensor insertion, leading to the possibility of allergic reactions [5]. These devices also require finger-pricking for calibration and in the cases of rapid fluctuations or unexpected symptoms. Such issues have made non-invasive monitoring a pressing priority to enable early detection and preventative treatment. Integrating various physical parameters and biomarkers could lead to the development of a reliable non-invasive blood glucose measurement system [6]. Devices estimating blood glucose using biofluids, such as tears, saliva, and sweat, are gaining attention in recent years; however, most are either expensive, complicated, or unreliable. Exhaled breath analysis is emerging as a promising methodology on account of its non-invasive nature, ease of sampling, and dynamicity.

Exhaled breath is a complex mixture comprising inorganic gases, non-volatile compounds [7], and more than 3500 VOCs [8]. Since the exhaled breath profile is enormously influenced by body metabolism, exhaled gases can serve as the biomarkers of diseases. The analysis of exhaled VOCs has drawn significant attention in recent decades in healthcare. Researchers have previously discussed the clinical potential of the exhaled VOC profile and have reviewed commercialized breath analyzers, and fabrication and detection methodologies, addressing the limitations of breath analysis [7,9,10,11,12]. Breath analysis can be broadly categorized as targeted or untargeted. The targeted approach is hypothesis-driven, whereas the latter is hypothesis-generating [13]. Targeted breath analysis is challenging because of the complex mixture of compounds present in the breath. On the other hand, non-targeted analysis requires complex statistical analysis of the generated data, where the lack of demographic representation creates bias, leading to reproducibility issues [13]. Irrespective of the adopted strategy, breath sampling and sensing constitute the foundation of a reliable analysis. Overlooking the sampled breath phase and confounding factors leads to inappropriate sampling, altering the sample’s actual composition. A poorly designed sensing unit may fail to detect exhaled VOCs present in sub-ppm/ppb concentration.

Although exhaled breath analysis is a promising methodology in non-invasive healthcare, identification of suitable biomarkers along with their efficient sampling and sensing remains a critical challenge. Additionally, the clinical implementation of breath analysis is limited, owing to the lack of standardization in procedures. This review critically analyses the developments and challenges in exhaled breath analysis for diabetes diagnosis and monitoring. First, we provide an overview of non-invasive diabetes monitoring devices that are either commercialized or in development. Next, we discuss the factors influencing the exhaled breath VOC profile and present potential standalone biomarkers and VOC clusters suitable for diabetes management. Finally, we highlight the recent buildout of breath-sensing methodologies, focusing on novel sensing materials and transduction mechanisms. The review concludes with a discussion on shortcomings and future directions for breath analysis.

## 2. Non-Invasive Diabetes Monitoring Devices

The non-invasive glucose-monitoring devices market is expected to increase by USD 11.35 million during 2021–2025 [14]. Table 1 lists some of the non-invasive glucose-monitoring devices under development or which are available in the market. MARD in Table 1 stands for Mean Absolute Relative Difference. It is the average absolute relative difference between the measured value and the value obtained through the reference device [15]. Demonstrating the proximity of measurements to correct values, MARD is an acceptable metric for assessing the performance of glucose-monitoring devices [15]. However, it is strongly influenced by the study’s design and should be perceived as a value with some uncertainty [16]. The studies conducting at-home analysis prefer using glucometers as a reference for performance assessment, whereas clinical studies predominantly use lab-based blood or plasma glucose measurements. Error grids are also used for gauging the performance of glucose-monitoring devices. They consider the potential clinical outcome of treatment based on the measurement method under study [15]. The Clarke Error Grid is one of such error grids. It consists of five distribution risk zones marked with the letters A to E, where each zone represents a clinical interpretation of device performance [15]. ISO 15197 is a standard with stricter accuracy criteria which was released in 2013 for glucose-monitoring and self-testing devices [15]. Notably, most non-invasive devices either use spectroscopy or some optical technique. GlucoTrack [17] and Egm1000™ [18] use ultrasound, thermal, and electromagnetic technologies together to counterbalance the demerits of each one individually. Most of the devices in Table 1 involve complicated technologies customized for a specific type of diabetes and patients of a particular age group.

**Figure 1:**
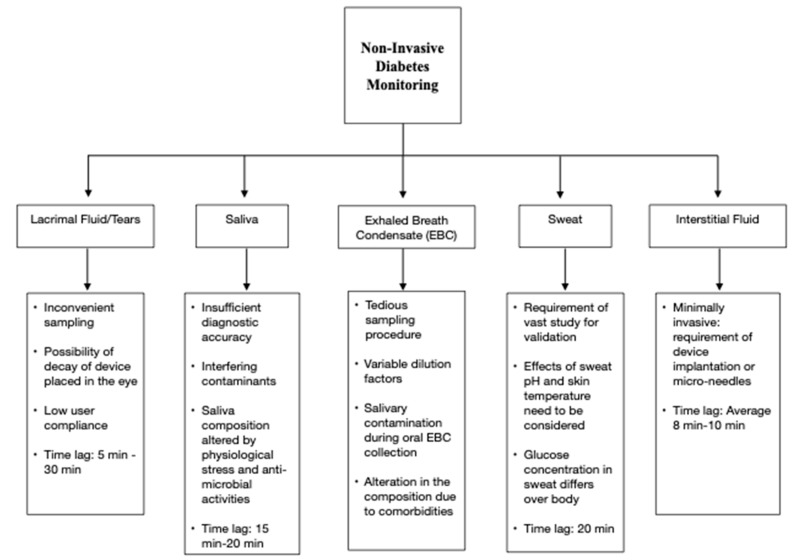
Non-invasive diabetes monitoring using biofluids.

Researchers have been focusing on the correlation between blood glucose and other biofluids such as sweat, saliva, tears, and interstitial fluid, which can be collected non-invasively or minimally invasively. However, the active body mechanisms minimizing glucose loss to these biofluids are a primary barrier to their utilization for non-invasive diabetes monitoring. Figure 1 summarizes the critical issues related to using these biofluids for glucose monitoring [6,19,20,21,22,23]. Saliva sampling is a relatively simple procedure, but it is prone to contaminations [6]. Tear-based analysis has low user compliance [20]. Blood glucose estimation using sweat requires developing proper sampling techniques [22]. Additionally, the glucose pathway from blood to sweat is yet to be deciphered [22]. Techniques targeting interstitial fluid sampling are usually minimally invasive but risk skin irritation [6]. Insufficient accuracy, sensitivity to environmental interferences, and time lag in measurements are other significant disadvantages associated with non-invasive monitoring approaches using biofluids.

**Table 1:** Glucose-monitoring devices working on non-invasive/minimally invasive technologies.

Technology	Device	Participants/Number of Paired Measurements	Performance	Measurement Area	Comments	References
NIR Spectroscopy	Wizmi	32 women 224 paired glucose measurements	MARD: 7.23%	Wrist	Patent-pending	[24]
Ultrasound + Thermal + Electromagnetic	GlucoTrack	91 subjects	MARD: 23.4% 97.3% readings lie in clinically acceptable zones in Clarke Error Grid	Earlobe	CE-certified Intended for adults (18 years or older) with T2DM or pre-diabetes Ear clip needs to be replaced after every six months Range of measurement: 70–500 mg/dL	[17,24,25]
Ultrasound + Thermal + Electromagnetic	Egm1000™	36 T2DM patients 11 people with prediabetes 188 paired glucose measurements	MARD: 13.8%	Earlobe	CE certified Intended for adults (18 years or older) with T2DM or pre-diabetes Compatible for 95% relative humidity Ear clip needs to be replaced after every 6 months Range of measurement: 70–500 mg/dL	[18,26]
Fluorescence	EverSense	23 subjects	MARD: 14.8%	Subcutaneous implant in the upper arm	Minimally invasive Suitable for 18 years or older adults 90 days lifetime of the sensor (FDA approved) On-body vibration alert for dangerous glucose swings	[27,28]
Reverse Iontophoresis	SugarBEAT	13,639 paired glucose measurements	MARD: 13.39%	Skin	CE certified Targets T2DM and prediabetes Sensor has to be disposed daily	[29,30,31]
Photo Thermal Detection	Diamontech D-Base	59 healthy subjects 41 subjects with diabetes	99.1% precise measurements	Finger	In development Fingertip to be relaxed on the sensor Suitable for all ages and both T1DM and T2DM	[32]
Tissue Photography Analysis	Tensortip Combo Glucometer	19 subjects	MARD: 17.1%	Finger	CE approved Has an add-on invasive glucometer	[33,34]
Subcutaneous Wired Enzyme Glucose Sensing	Abbott FreeStyle^^®^^ Libre	144 subjects	MARD: 9.2%	Upper arm skin (Sensor uses thin filament inserted just under the skin)	FDA-cleared Suitable for age four years and above people Minimally Invasive	[35,36,37]
Radio Wave Spectroscopy	Glucowise™	N/A	N/A	Skin between thumb and forefinger or earlobe	In development	[38]
Infrared Spectroscopy	Tech4Life Enterprises Non-Invasive Glucometer	N/A	N/A	Finger	In development	[39]
Photoplethysmography	HELO Extense	N/A	N/A	Finger	Certified as Medical Device Class 1 for user safety in Europe Not targeted for diabetes but general sugar trend monitoring	[40]
MIR spectroscopy/Optical Parametric Oscillation	Light Touch Technology	N/A	99% of measured values are within A zone and B zone defined by the ISO 15197 standard	Hand	In development	[41]
SkinTaste Technology: Biosensors and array of micropoints	K’Watch Glucose	N/A	N/A	Wrist	Uses a hypo-allergic pad that requires replacement after seven days Minimally invasive In development	[42]
Radiofrequency Sensor Technology	Alertgy	N/A	N/A	Wrist	In development For T2DM	[43]
Bio RFID Technology: Spectroscopy	UBAND-Know Labs	N/A	4.3% mean difference compared to FreeStyle Libre	Wrist	In development	[44]
Photoplethysmography	LifePlus: LifeLeaf	N/A	N/A	Wrist	Patent-pending	[45,46]
Tear Sensor	Noviosense	24 T1DM subjects	MARD = 16.7%	Lower Eyelid	Targeted for T1DM	[47,48,49]
Sensors based on photonics sensing technology	Indigo Diabetes	N/A	N/A	Subcutaneous implant	Minimally invasive In development	[50]

## 3. Potential Breath Biomarkers of Diabetes

**Figure 2:**
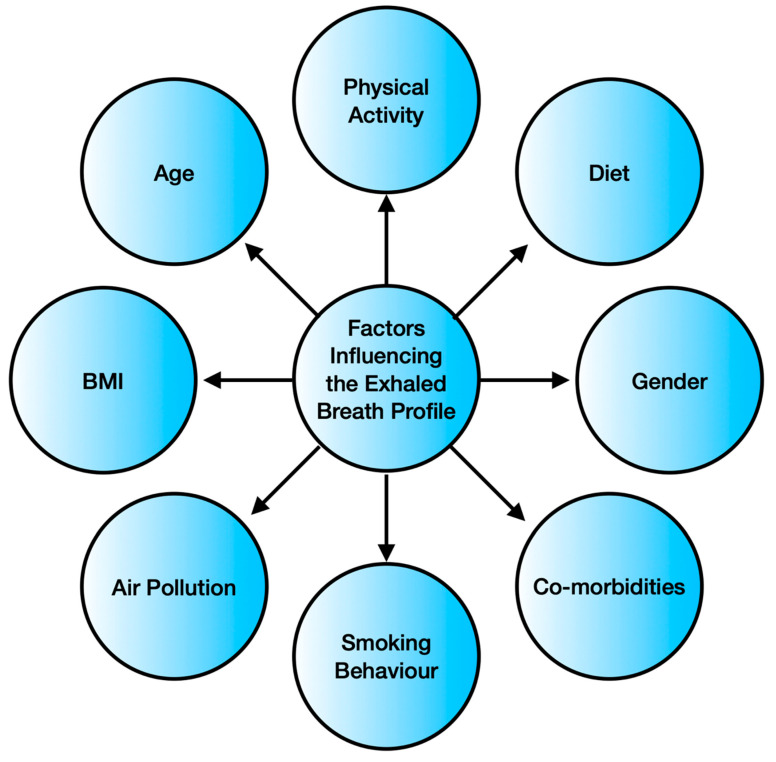
Factors influencing the exhaled breath profile.

Breath analysis is emerging as a popular non-invasive disease-monitoring tool, owing to the easy accessibility and simpler nature of the breath sample matrix in comparison to the serum/urine matrix [51]. Its user-friendliness and point of care operation are the added advantages, making breath-monitoring a potential strategy for non-invasive health management [52]. However, the composition of exhaled breath depends upon various factors, as indicated in Figure 2.

**Figure 3:**
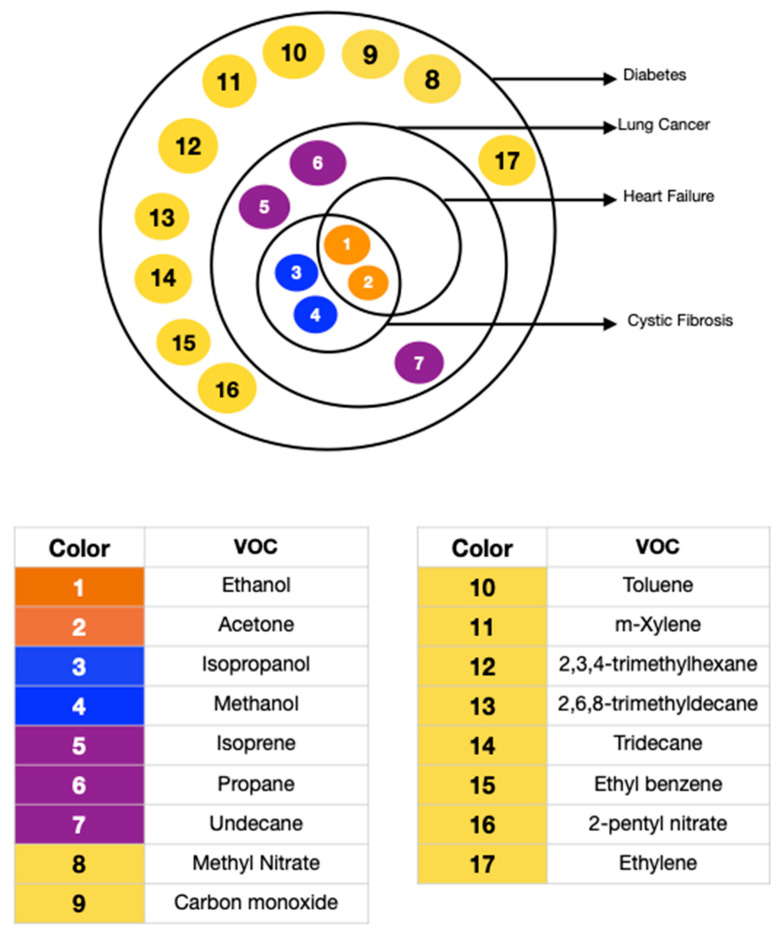
Breath Biomarkers associated with Diabetes (8–17 yellow) and overlapping with Lung Cancer (5–7 purple), Heart Failure (1–2 orange), and Cystic Fibrosis (1–2 orange and 3–4 blue).

Physical activity causes physiological changes leading to higher O_2_ requirements, increased blood pressure, variation in blood pH, and alteration in systemic oxidative stress [53,54]. These are well-reflected in the exhaled volatiles. Diet directly impacts the metabolism as well as the gastrointestinal flora. A high-fat diet has been observed to increase the level of expired NO [55]. A ketogenic diet can raise the exhaled breath acetone to even five times in a healthy subject [55]. Exposure to aromatic compounds, sulfur compounds, and other air pollutants is also a prominent exogenous factor determining the nature of exhaled breath. The inhaled compounds may get subjected to metabolic functions, leading to an unexpected inhale/exhale concentration ratio. A study consisting of 1417 adults confirmed that the body mass index (*p*-value < 0.001), age (*p*-value = 0.01), gender (*p*-value < 0.001), and smoking habits (*p*-value < 0.001) significantly influence the exhaled breath content, with smoking being the dominating factor [56]. The presence of comorbidities complicates the exhaled breath analysis further. Diabetes is often accompanied by other health conditions, such as kidney diseases, diabetic ketoacidosis, diabetic foot, obstructive sleep apnea syndrome, halitosis, and *Helicobacter pylori* infection [57]. Not only do numerous diseases co-exist, but they may also have similar breath biomarkers. Yokokawa et al. conducted a study including 35 diabetes patients with stage C heart failure and 20 diabetes patients with or at risk of heart failure (stage A or stage B). They concluded that people with both stage C heart failure and diabetes exhale higher amounts of acetone [58]. Table 2 lists and Figure 3 pictorially presents the diseases that have biomarkers overlapping with diabetes.

### 3.1. Standalone Breath Biomarkers of Diabetes

**Figure 4:**
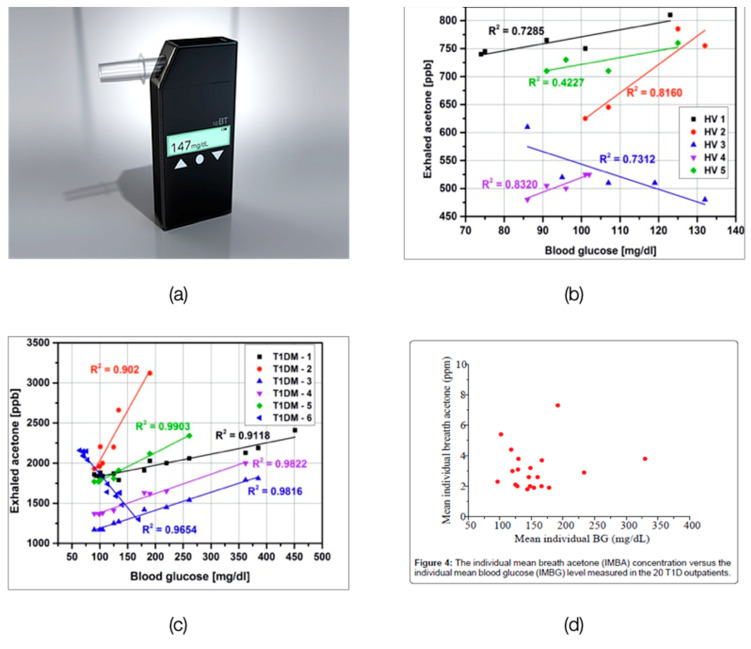
(**a**) Glucair: Pain-Free Diabetic Glucose Breath Detector. Reprinted with permission from Breath Health Inc. [67] (**b**,**c**): Relation between breath acetone as measured by mass spectrometry and blood glucose (**b**) Healthy Volunteers [68] (**c**) TIDM subjects [68] (**d**) The individual mean breath acetone concentration versus the individual mean blood glucose (IMBG) level measured in the 20 T1DM outpatients (no strong correlation) [69].

Numerous researchers have tried to correlate diabetes with a single targeted exhaled VOC. Table 3 lists these potential standalone biomarkers of diabetes. A higher level of breath acetone (T2DM: >1.71 ppm, T1DM: ≥2.19 ppm, can go up to 21 ppm [62,63,64]) is hypothesized to be an indicator of diabetes as the insulin in the body inhibits ketone synthesis, and insulin levels are generally low in people with diabetes. Breath Health, Inc. is developing a pain-free diabetic glucose breath detector based on exhaled breath acetone detection (Figure 4a). The device comprises single-use sensor slides made from polymer films of 4-vinyl benzene boronic acid and allylamine hydrochloride that can react with the acetone in the exhaled breath via petasis reaction [65]. However, the relevance of acetone as the sole biomarker for diabetes-monitoring is uncertain, as most single measurement studies report no correlation between exhaled acetone and blood glucose, whereas continuous monitoring studies report both positive and negative correlations [66]. (Figure 4b–d).

Likewise, the correlation of exhaled ethanol and blood glucose is also debatable due to contrasting observations [70,71]. Exhaled isoprene, carbon monoxide, methyl nitrate, pentanal, isopropanol, and dimethyl sulfide levels are higher in T1DM patients [72,73,74,75]. T2DM patients exhale elevated amounts of isopropanol, ethylene, ammonia, carbon monoxide, toluene, 2,3,4-trimethylhexane, 2,6,8-trimethyldecane, tridecane, and undecane, but lesser m-xylene [62,72,76,77,78,79]. However, these results are based on studies including cohorts not large enough to encompass all the determining factors. Thus, a suitable standalone biomarker is yet undiscovered.

**Table 3:** Potential Standalone Exhaled Breath Biomarkers of Diabetes.

Type of Diabetes	Potential Breath Biomarkers	References
T1DM	Acetone Ethanol Carbon Monoxide Isoprene Propane Methyl Nitrate Pentanal Isopropanol Dimethyl Sulphide	[10,63,70,72,73,75,76,80,81]
T2DM	Acetone Isopropanol Ethylene Ammonia Carbon Monoxide Toluene m-Xylene 2,3,4-trimethylhexane 2,6,8-trimethyldecane Tridecane Undecane	[62,72,76,77,78,79]

### 3.2. Breath Biomarker Clusters of Diabetes

The lack of one-to-one correspondence between the exhaled VOCs and diseases, and the dynamic nature of the exhaled breath profile has led to more studies focusing on clusters of compounds to ensure the inclusion of comorbidities and intra-individual variabilities [10]. Table 4 gives a brief outline of such studies and their deductions. Minh et al. used gas chromatography to identify about 100 VOCs in the exhaled breath [82]. They observed a strong correlation between glucose measurements and the estimates made using a cluster of VOCs consisting of acetone, methyl nitrate, ethanol, and ethylbenzene and another cluster of 2-pentyl nitrate, propane, methanol, and acetone. Mansouri et al. employed a three-gas prediction model for blood glucose prediction [83]. They observed that the actual blood glucose values correlate with those estimated using different possible combinations of the three exhaled gases, namely, ethanol, acetone, and propanol. The best results were obtained for multiple linear regression, considering all three gases together. Yan et al. determined eight potential T2DM breath biomarkers using the gas chromatography–mass spectrometry (GC-MS) technique [79]. They observed that a cluster containing isopropanol, 2,3,4-trimethylhexane, 2,6,8-trimethyldecane, tridecane, and undecane could be used as a predictive biomarker group for clinical diagnosis.

The strategy of using a VOC cluster for diabetes breath diagnostics is a promising development in exhaled breath analysis for healthcare. However, the results from various studies cannot be combined as researchers use different breath sampling and analysis techniques in their studies. Moreover, the factors such as the diet of the subjects, time between meals and tests, medication, insulin injection, and medical history introduce drastic variations in the collected auxiliary data. Thus, wide-scale research with standardized methodologies is needed to deduce the best cluster for diabetes management, suitable for diverse groups of people.

## 4. Sensing Methodologies for Breath Analysis

Effective breath analysis requires a sensing unit with high sensitivity and selectivity, low limits of detection, adequate stability, rapid detection, and a convenient user interface [84]. This section sheds light on the recent developments in exhaled breath sensing, focusing mainly on the sensing materials and transduction mechanisms.

Spectrometry-based techniques constitute the standard exhaled breath analysis methods, and most breath biomarkers to date have been identified using them, owing to their reliability, high sensitivity, and low detection limit [12,85]. Gas chromatography (GC) is the gold standard for VOC identification, but its operation demands expertise and is bulky and expensive [9]. Similarly, other spectrometry-based techniques also have associated disadvantages. Ion Mobility Spectroscopy (GC-IMS) is inapt for unknown compound identification [12]. Proton Transfer Reaction Mass Spectrometry (PTR-MS) and Selected Ion Flow Tube Mass Spectrometry (SIFT-MS) lack specificity, require a skilled operator, and are unsuitable for molecules with low proton affinity [12]. Though spectrometry-based techniques are apt for offline analysis in hospitals or diagnostic clinics, the above-listed drawbacks have led to research on alternate sensing methods to develop a portable, compact, and user-friendly diabetes management system.

The sensors used in breath analysis can be classified into chemiresistive, electrochemical, optical, piezoelectric (mass-sensitive), and several other categories based on the involved transduction mechanism. A transducer is responsible for generating measurable signals from the analyte’s interaction with the sensor and, therefore, is a determining factor for reliable measurement.

### 4.1. Chemiresistive Sensing

Chemiresistive sensing is an emerging non-invasive healthcare technique due to its high sensitivity, compactness, cost-effectiveness, portability, and ease of fabrication [86]. These sensors rely on changes in electrical conductivity caused by an interaction with the analyte. Metal oxide semiconductors, carbon nanotubes, graphene oxides, metal chalcogenides, and conductive polymers are amongst the most popular chemiresistive materials.

#### 4.1.1. MOS Sensors

**Figure 5:**
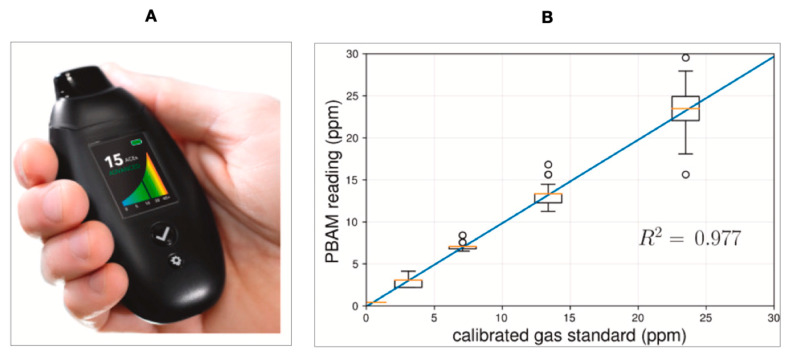
(**A**) PBAM developed by Readout Health [87]; (**B**) Performance of three calibrated PBAM’s against a laboratory gas standard [87]. Reprinted with permission from the BIOSENSE.

Metal oxide semiconductor (MOS) sensors are extensively used in breath sensing. The small size, ease of operation, inexpensiveness, and low maintenance make MOS sensors one of the best candidates for breath analysis [8]. BIOSENSE™ Readout Health has developed a high-resolution portable breath acetone meter (PBAM) (Figure 5A,B) based on chemiresistive metal oxide semiconductor (MOS) sensors [87]. Keyto is another reported device based on the nanostructured semiconducting metal oxide core selective to acetone [88].

**Figure 6:**
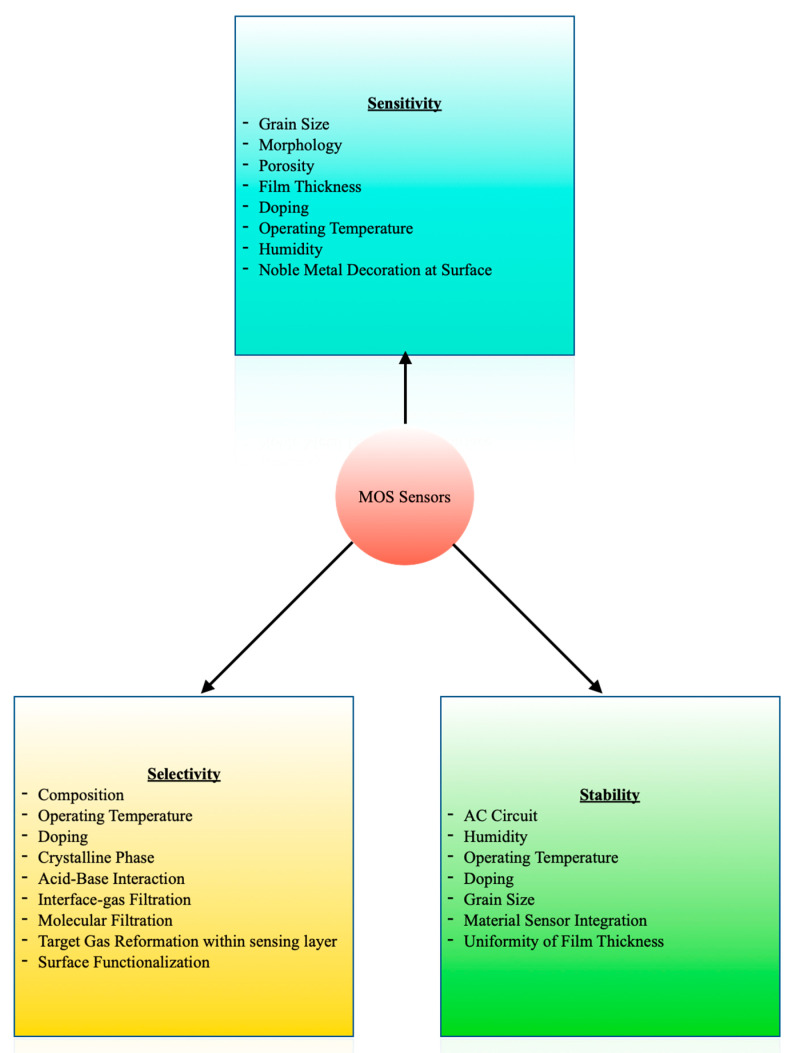
Parameters Affecting the Sensing Performance of MOS Sensors.

The sensitivity, selectivity, and stability of the MOS sensors are determined by several factors [8,89], as indicated in Figure 6. This multi-factor dependence provides liberty to tailor the sensors as per the requirement of the application. Power consumption, heat generation, lack of selectivity, and humidity interference are the most common issues associated with MOS sensors [8]. There has been research tackling these problems and selecting suitable materials for the fabrication of sensors satisfying the requirements of an inexpensive portable device.

Cheng et al. used planar MEMS technology to develop a MOS gas sensor with SnO_2_ as the sensing material. The operating temperature was 400 °C, and the sensor required a microheater, consuming 39 mW of power. The sensor was found adequately sensitive to 1 ppm ethanol [90]. Siebert et al. introduced a mixed semiconducting metal oxide sensor selective to acetone with the highest response of 50% at an operating temperature of 300 °C. The printed Cu and Fe microparticles were annealed at 425 °C in the air for 4 h, leading to highly porous bridging non-planar CuO/Cu_2_O/Cu—Fe_2_O_3_/Fe nanostructures beneficial for sensitive detection. The lowest power consumption was about 0.26 µW for 100 ppm acetone [91].

Das et al. prepared a highly sensitive, fast, and stable (negligible change in base resistance for at least six months) cobalt chromite thick film-based trace acetone sensor [92]. It showed minimal cross-sensitivity to ethanol, ammonia, and saturated moisture. The response to one ppm, two ppm, and five ppm acetone was 3.81, 4.82, and 6.64 folds, respectively, and thus, a clear resolution existed between lower concentrations of acetone. Hanh et al. utilized the synergetic effect of the hollow structure of Zn_2_SO_4_ (ZTO) and the high catalytic activity of the Pt catalyst to develop a highly sensitive and stable acetone sensor with a limit of detection at the ppb level [93]. The Pt10-ZTO sensor, that is, the sensor with a Pt loading amount of 1 wt%, performed best amongst the sensors with different compositions. Brahma et al. reported the enhanced sensitivity and specificity to acetone at room temperature on doping p-type ZnO with Cu [94]. In contrast, no such response was observed for the n-type undoped and n-type Cu-doped ZnO. Kim et al. prepared a SnO_2_ nanosheet gas sensor with mainly (101) crystal facets exposed [95]. The nanosheets synthesized for 6 h (NS-6) had the highest sheet area, leading to a 10 times higher response than those synthesized for 2 h and 24 h. They concluded that controlling the crystal facet of a nanomaterial can enhance the sensing characteristics without the requirement of noble metal decoration. Xu et al. synthesized WO_3_ nanofibers using SiO_2_ nanoparticles and polyvinylpyrrolidone (PVP) as sacrificial templates, ammonium paratungstate as a tungsten precursor, and water as a solvent [96]. WO_3_ nanofiber-based sensors were found to show magnificent acetone-sensing capabilities with a low detection limit, fast response and recovery, and high stability. The uniform mesopores assisted the diffusion of gas molecules, and the highly crystalline nature also supported the rapid transportation of charge carriers to the bulk. The abundant active sites and high specific area contributed to the large adsorption of acetone molecules. Table 5 contains the specifications of some recently developed sensors selective to acetone. As evident from the table, the rapid response and recovery time makes these sensors compatible for real-time analysis of exhaled breath. Several other reviews focus on MOS sensors for exhaled breath analysis [11,89,97].

#### 4.1.2. Other Chemiresistive Materials

Carbon nanotubes (CNTs), graphene, and semiconductor chalcogenides are also being studied for breath-biomarker detection [8]. Freddi et al. developed a sensing array for human breath analysis based on Single-Walled Carbon Nanotube (SWCNT) layers functionalized with semiconductor organic molecules. It was found that the sensor array could detect target gases with a clear fingerprint [98]. Liu et al. proposed a graphene quantum dot (GQD) functionalized three-dimensional ordered macroporous (3DOM) ZnO structure. The GQD-modified 3DOM-structured ZnO sensor displayed a rapid and high response and good selectivity towards acetone. It could differentiate the exhaled breath of healthy people and those with diabetes [99]. ZnS, a transition metal chalcogenide, has interstitial defects, trapped surface states, and sulfur vacancies, facilitating oxygen adsorption crucial for gas sensing [100]. It further has commendable electrical, optical, and catalytic properties. Mishra et al. used a hot injection method to synthesize ZnS quantum dots (QDs) for acetone detection [100]. The ZnS QDs sensor’s optimal operating temperature was reported to be 175 °C. The sensitivity and selectivity to 100 ppm acetone at 175 °C were 92.4% and 91.1%, respectively. The theoretical detection limit was found to be 1.2 ppm. In addition, the sensor exhibited quick response and recovery (5.5 s and 6.7 s, respectively). Organic material-based sensors are also gaining popularity due to their small size, low cost, and room temperature operation [101]. Chuang et al. developed a room-temperature-operating poly[(9,9-dioctylfluorenyl-2,7-diyl)-co-(4,4′-(N-(4-sec-butylphenyl)diphenylamine)] (TFB) acetone sensor based on cylindrical nanopore structures [101]. It exhibited a response of 5% to 300 ppb of acetone. The TFB sensor was also sensitive to ammonia. Hence, the authors designed a filter utilizing the water solubility of ammonia to improve acetone selectivity. Sensors based on CNTs, graphene, and semiconductor chalcogenides usually operate at lower temperatures than the MOS sensors. CNTs are minimally sensitive to moisture due to the hydrophobic surfaces [86]. Graphene demonstrates excellent sensing due to a higher theoretical specific surface area [86]. Semiconducting chalcogenides can be used to detect both polar and nonpolar gases [8]. These distinct properties have opened avenues for developing different types of chemiresistive sensors, rather than relying on only metal oxides.

### 4.2. Electrochemical Sensing

**Figure 7:**
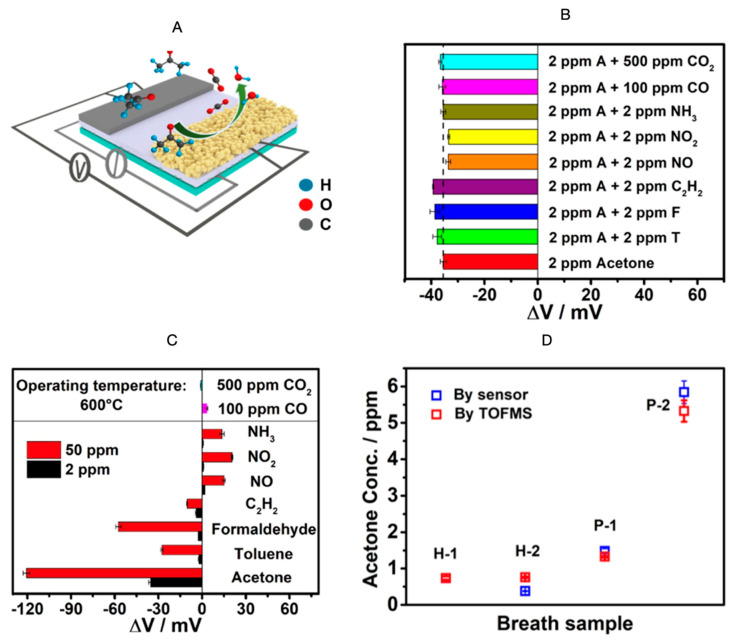
(**A**) Schematic diagram of measurement; (**B**) Cross-sensitivities of the sensor to the gas mixtures of 2 ppm acetone and other interference gases; (**C**) Selectivity of the sensor to various gases at 600 °C; (**D**) Acetone concentration in the breath sample calculated by the sensor and tested by TOFMS (H: Healthy; P: Patient) [103]. Reprinted with permission from Elsevier.

Electrochemical sensors have been gaining attention in the breath analysis area due to their highly selective nature, low cost, miniaturizability, low power requirement, and biocompatibility [8]. Lavanya et al. reported the utility of a Zn-MgNi_2_O_3_ conductometric sensor, selective to acetone and with a 0.5 ppb limit of detection [102]. Zn-MgNi_2_O_3_ was also found suitable for electrochemical detection of glucose. Jiang et al. fabricated an yttria-stabilized zirconia (YSZ)-based mixed potential acetone sensor using a Cd_2_SnO_4_ sensing electrode [103]. Figure 7A depicts the schematic of the sensor measurement setup. The sensor was found to be negligibly cross-sensitive to other gases (Figure 7B), but highly selective to acetone (Figure 7C). Moreover, there was no significant difference between the exhaled breath acetone measurement values obtained by the sensor and those obtained using time-of-flight mass spectrometry (TOFMS) (Figure 7D). Operating at a temperature of 600 °C, the sensor’s response value to 10 ppm acetone exhibited a slight fluctuation (±7%) after consecutive high-temperature measurements of more than 75 days. The detection limit was reported to be 50 ppb. The sensor also demonstrated a satisfactory humidity resistance. The response to 10 ppm acetone did not vary substantially in the relative humidity range of 60% to 98%.

Liu et al. also fabricated a mixed-potential-type acetone sensor based on Ce_0.8_Gd_0.2_O_1.95_ (GDC), with a detection limit of 0.3 ppm and an operating temperature of 590 °C [104]. The sensor was found to be stable in 45 days of continuous testing. The authors suggested using a dehumidifier to remove moisture, adding a hydrophobic layer to modify the sensing layer, or including a humidity compensation device in the system to reduce the influence of humidity on the measurements.

### 4.3. Piezoelectric Sensors

**Figure 8:**
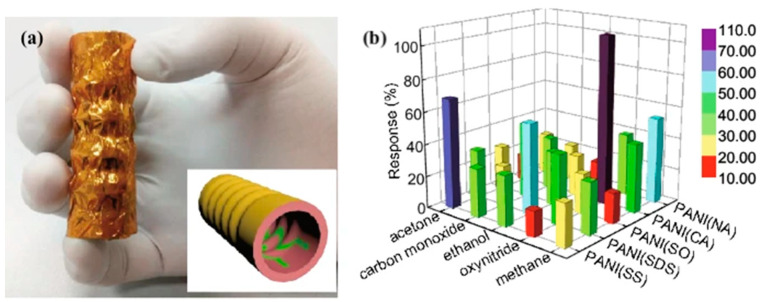
(**a**) PANI/PVDF bellow; (**b**) The response of the five sensing units to 600 ppm gases at room temperature [105] (Link to the Creative Commons License: http://creativecommons.org/licenses/by/4.0/ (accessed on 14 October 2021)).

Some studies also report the usage of piezoelectric sensors for exhaled breath sensing. Fu et al. developed a self-powered breath analyzer based on polyaniline/polyvinylidene fluoride (PANI/PVDF) piezo-gas-sensing gas arrays [105]. Figure 8a shows the PANI/PVDF bellow comprising five PANI/PVDF electrodes. Each PANI derivative was doped with an individual dopant and was labeled accordingly. PANI (SS), PANI(SDS), PANI(SO), PANI(CA), and PANI(NA) correspond to the following dopants, respectively: sodium sulfate, sodium dodecylbenzene sulfonate, sodium oxalate, camphorsulfonic acid, and nitric acid. The device worked at room temperature by converting exhaled breath energy into electrical signals without external power sources. The gas markers of PANI(SS), PANI(SDS), PANI(SO), PANI(CA), and PANI(NA) were reported to be acetone, ethanol, CO, NOx, and CH_4_, respectively. Since each sensing unit demonstrated selectivity to a specific gas, the device was proposed for multiple disease diagnoses. The response of five sensing units to 600 ppm of different gases is shown in Figure 8b. The gas flow rates did not influence the sensors’ response, making them suitable for use in exhaled breath analyzers.

### 4.4. Optical Sensing

**Figure 9:**
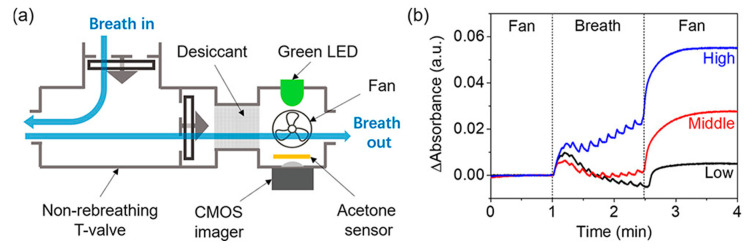
(**a**) Schematic of a homemade device based on a colorimetric sensor for breath acetone (BrAce) detection. (**b**) Signals of three BrAce tests with different acetone concentrations. Reprinted with permission from [108]. Copyright (2021), American Chemical Society.

The interaction between an analyte and a biorecognition substance results in optical changes measurable by colorimetric, fluorescence, chemiluminescence, or scattering mode [7]. Ye et al. developed a fiber-optic biochemical acetone sensor using a flow-cell with a nicotinamide adenine dinucleotide (NADH)-dependent secondary alcohol dehydrogenase (S-ADH) immobilized membrane attached to a fiber-optic NADH measurement system [106]. UV-LED with a peak emission of 335 nm was used as an excitation source. The relationship between acetone concentration from 20 ppb to 5300 ppb and fluorescence was established. The sensor exhibited a response time of 35–70 s corresponding to 95% of the steady state. Chien et al. also came up with a bio-sniffer, utilizing the NADH fluorescence as the signal, targeting isopropanol in the exhaled breath [107]. The detection limit was reported to be 0.5 ppb. The humidity in the sample had a negligible effect on the measurements. Wang et al. reported a colorimetric sensor for breath acetone detection using a reaction between acetone and hydroxylamine sulfate [108]. Figure 9 shows a schematic of the device and a plot of signals of breath acetone tests. The designed sensor was disposable and did not require frequent calibration.

Colorimetric sensing has been extensively used for lung cancer diagnosis using exhaled breath. Instead of using a single colorant, researchers opt for a larger number of colorants belonging to different chemical categories to accommodate a maximum number of VOCs and observe the change in microarray with the subject’s health condition [109]. The same approach can be used for diabetes monitoring as well. Optical sensors are receiving attention for breath sensing, but they are prone to environmental interferences and sometimes require complete isolation to alleviate external light. Thus, these sensors require further improvements in terms of the stability of the sensing system [7].

### 4.5. FET Sensing

**Figure 10:**
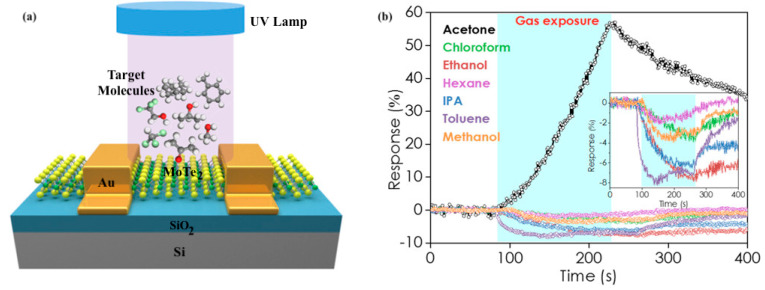
(**a**) Schematic diagram of MoTe_2_ FET sensing setup; (**b**) Sensing response of the MoTe_2_ FET toward seven different VOCs, including acetone, chloroform, ethanol, hexane, isopropanol, toluene, and methanol, all at 100 ppm. Measurements were taken under UV light. Only acetone induced a positive response (increase in conductance upon gas exposure). Reprinted with permission from [114]. Copyright (2018), American Chemical Society.

Field Effect Transistor (FET)-type gas sensors are known for their small size, low power consumption, and high stability [110,111]. Nanomaterials such as carbon nanotubes, nanowires, graphene, and transition metal chalcogenides are used to enhance their properties [112]. Yu et al. developed a gas-sensitive field-effect transistor with ZnO nanorods for non-invasive diabetes detection at room temperature [113]. The target analyte was acetone, and the detection limit was found to be 0.8 ppm. Wu et al. achieved highly sensitive and selective acetone detection through MoTe_2_ FETs under UV illumination [114]. The schematic diagram of the MoTe_2_ FET sensing setup is given in Figure 10a. The sensing response to 100 ppm of seven gases, namely, acetone, chloroform, ethanol, hexane, IPA, toluene, and methanol, was negative in the dark. UV light illumination led to a positive response to acetone, but no change was observed for other VOCs (Figure 10b). The transformation of acetone from a weak reducing agent to a weak oxidizing agent due to the UV absorption and intramolecular photon–electron interaction promoted by the acetyl group was recognized as the possible reason for this unique behavior. Further, the sensor response was also positive for other ketones, including 2-pentanone and 3-pentanone. UV-assisted sensing also improved the detection limit of the MoTe_2_ FET gas sensor from 23 ppm in the dark to 200 ppb. Along with sensitivity and specificity to acetone, the sensor demonstrated stability to humidity. The response to 100 ppm to 2000 ppm acetone at 45% and 65% relative humidity, respectively, was independent of the humidity level.

### 4.6. Wearable Sensing

There has been a gradual shift from a hospital-centered health monitoring system to individual-centric healthcare owing to the increasing population, health issues, and associated economic burdens. Wearable technologies are paving the way for personalized healthcare as they can provide real-time, continuous, and fast detection of biomarkers from the human body. The lightweight, low-cost, and commendable stretchability of flexible sensors make them an ideal platform for biosensing and wearable bioelectronics [115]. These sensors can be further classified as “on-body”, “in-clothing”, and “accessories’’-type sensors. The “on-body- type sensors are attached to the surface of a body part. The “in-clothing” sensors are integrated with wearable textiles, and the “accessories’’-type sensors are usually made a part of wristwatches, wristbands, rings, and armbands [116].

**Figure 11:**
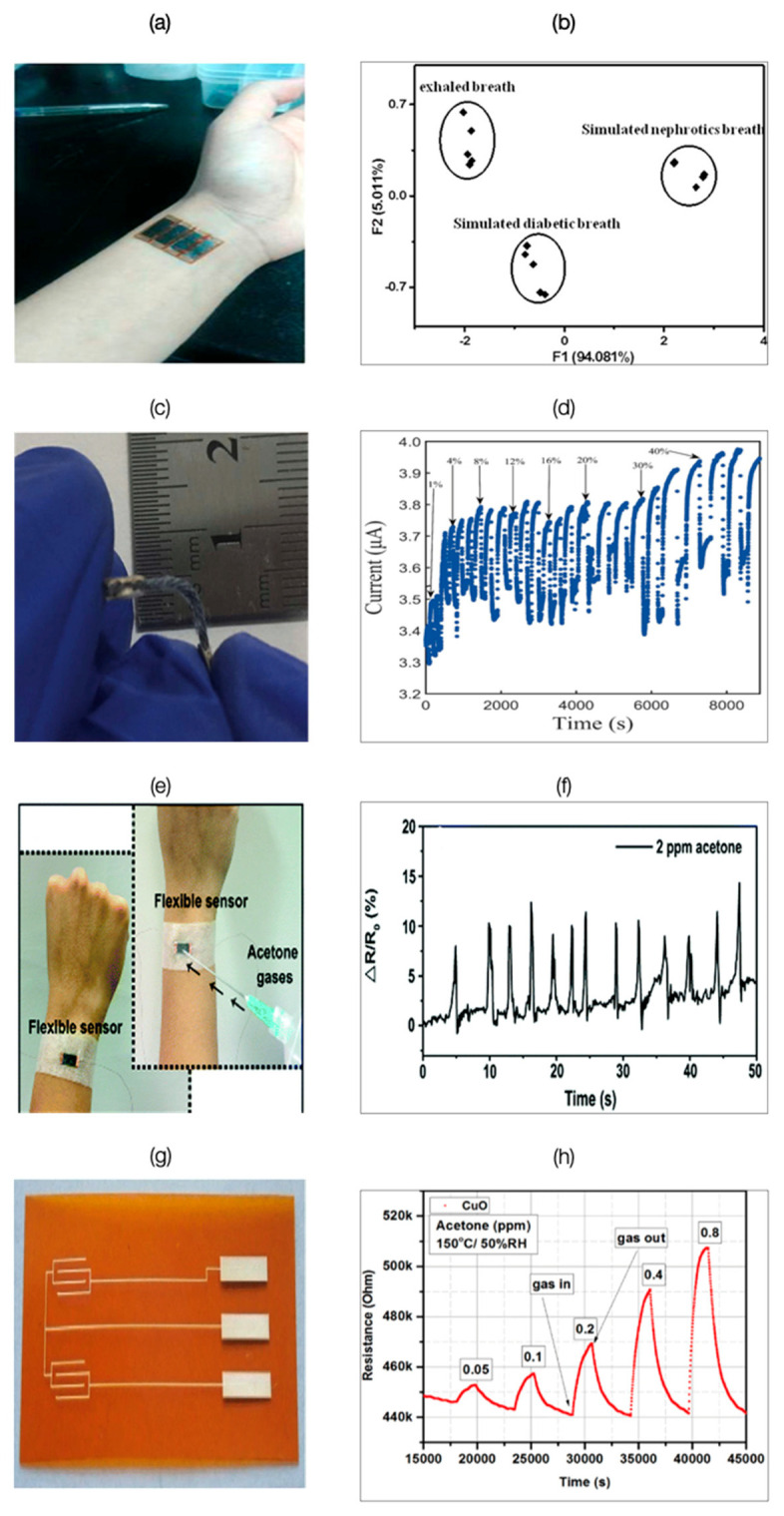
(**a**,**b**): On-body Sensor (**a**) Photograph of the as-prepared multifunctional wearable device mounted on the human wrist for simultaneously monitoring VOC-related disease; (**b**) PCA of exhaled breath of simulated nephrotics patients, diabetic patients, and healthy people. Reprinted with permission from [117]. Copyright (2018), American Chemical Society. (**c**,**d**): In-clothing type sensor; (**c**) Camera image of a cotton thread with PEDOT:PSS; (**d**) Signal–response curve to acetone at different concentrations. Sensing experiment for each concentration is repeated three times with the sensor (n = 3). Reprinted with permission from [118]. Copyright (2016), The IOPscience; (**e**) Photograph of a proof-of-concept wearable sensor and illustration of the performance evaluation method. (**f**) Real-time sensor response to a pulsated ejection of simulated diabetic breath containing 2 ppm of acetone vapor (85% RH) flown directly over the sensor. Reprinted with permission from [119]. Copyright (2013), The Royal Society of Chemistry; (**g**) Resistance changes for Kapton-based CuO gas sensor; (**h**) General view of the electrode layer [120] (Link to the Creative Commons License: http://creativecommons.org/licenses/by/4.0/ (accessed on 14 October 2021)).

Xu et al. fabricated a multifunctional wearable sensing device (Figure 11a) based on two graphene films for simultaneous detection of physiological signals and VOCs [117]. The sensor array integrated with pattern recognition detected and discriminated eight different VOCs, namely ethanol, ethyl acetate, dichloromethane, acetaldehyde, isopropanol, acetone, ammonia, and methanol. Principal component analysis (PCA) of the exhaled breath of five healthy subjects and that of five simulated exhaled breaths of subjects with diabetes and nephrotic disease led to distinguishable clusters without any overlap (Figure 11b). Zhang et al. developed a PEDOT:PSS sensor based on a cotton thread [118]. PEDOT:PSS acted as a chemical resistor and underwent conductivity changes on exposure to acetone. The sensor was found to be flexible enough for incorporation in clothing and other wearables (Figure 11c). The sensor’s current measurements were observed to be proportional to acetone concentration, but the response became indistinguishable after a certain level (Figure 11d). Additionally, the sensor was only found suitable for acetone concentrations producing signals above 3%, as water vapors also generated an average signal of 3%. The washing experiments conducted on the sensor indicated no significant variation in performance for a washing duration up to 20 min and heating durations of up to 30 min, respectively. These results attest to the possibilities of developing sensors integrated with textiles. Wang et al. developed an acetone sensor by depositing chitosan and reduced graphene oxide (CS-rGO) biocomposite on mechanically flexible cellulose paper (Figure 11e) with a response time ≤ 1 s [119]. The sensor’s response to simulated breath containing two ppm acetone is given in Figure 11f. The limit of detection was estimated to be 20 ppb. Andrysiewicz et al. reported a flexible Kapton-based CuO gas sensor (Figure 11g) operating at a temperature of 150 °C [120]. It demonstrated a detection limit of 0.05 ppm. The changes in the sensor’s resistance on exposure to acetone ranging from 0.05 ppm to 0.8 ppm concentration are given in Figure 11h. Though the developed sensors exhibited characteristics compatible with a portable breath analyzer, they require humidity compensation due to their sensitivity to moisture.

The rapid progress in the development of flexible sensors is apparent. However, along with a suitable sensing system, the issues related to power management, real-time communication, system integration, data security, calibration, and biocompatibility have to be mitigated to devise feasible wearable devices [115,121]. Zou et al. reviewed the possibilities of human body energy harvesting for bioelectronic devices and concluded that chemical, mechanical, and thermal energy from the human body could be utilized for powering smart bioelectronics, including wearable devices [122]. Xue et al. developed a self-powered breathing and temperature sensor by mounting a metal-coated PVDF film with electrodes on an N-95 respirator, forming a pyroelectric nanogenerator [123]. The pyroelectric generator was found to generate an open-circuit voltage of 42 V and a short-circuit current of 2.5 μA.

## 5. Discussion

As per a report by the International Diabetes Federation (IDF), there will be around 700 million people with diabetes by 2045 [1]. Non-invasive diabetes monitoring solutions are urgently required to enable self-health management, precision medicine, and telemedicine, but there is still no concrete information about the breath biomarkers associated with diabetes. Most of the studies focusing on diabetes monitoring through exhaled breath revolve around exhaled acetone. However, a better perspective would be identifying the clusters of biomarkers of which acetone, perhaps, can be the key component. The specificity of the sensing unit cannot be put to use until the target is identified. A generic approach is to use cross-reactive sensors along with the pattern-recognition systems, that is, an electronic nose, but it assists in identifying the fingerprints of a disease and not the specific breath biomarkers. Sarno et al. developed an electronic nose using a deep neural network to classify people into three categories, namely, healthy (Blood Glucose (BG) < 120 mg/dL), suffering from prediabetes (BG: 120–150 mg/dL), and diabetes (BG > 150 mg/dL) [124]. It consisted of a temperature-humidity sensor and four MOS sensors, with the main target biomarkers being carbon dioxide, carbon monoxide, acetone, and other VOCs, respectively. The study included 10 people belonging to each category. The system achieved an accuracy of 96.29% and an error rate of 0.050. Bahos et al. tested an electronic nose comprising a surface acoustic wave (SAW) sensor array based on zeolitic imidazolate frameworks, ZIF-8, and ZIF-67 nanocrystals (pure and combined with gold nanoparticles) as sensitive layers, to detect acetone, ethanol, and ammonia [125]. The SAW sensor array consisted of four sensing layers with different compositions and performance characteristics. The system operated at room temperature and demonstrated biomarker discrimination ability. Wulandari et al. reviewed the electronic noses for diabetes detection. They reported the recent progress in sensor types, number of sensors, characterization methods, feature extraction methods, and pattern recognition methods used in an electronic nose [126], concluding that there has been a significant development in characterization methods, but feature extraction and pattern recognition have not advanced much in the past few years.

The integration of smartphones with exhaled breath sensors is emerging as a constructive implementation approach for personal healthcare. It simplifies the analysis due to smartphones’ high computational abilities and image-recognition features. Shreshtha et al. proposed a microcontroller-based solution to classify low (100 mg/dL or lower) and high blood glucose (125 mg/dL and higher) levels using ethanol and acetone as the biomarkers [127]. The trained vector machine algorithm could perform the classification with 97% accuracy. They also developed a wearable smart wristband platform consisting of three MOS sensors, an Arduino-based Adafruit FLORA Microcontroller, GPS module, LED light, rechargeable Li-ion battery, and a Bluetooth module. These components enabled data collection, transmission to a smartphone app, and pushing the data into the cloud for analysis. A cancer diagnosis device, SniffPhone, that allows patients to exhale into a mouthpiece that is attachable to a smartphone and get instantaneous results is under development. It consists of an array of nano-material-based chemical sensors and integrated on-chip microfluidics and electronics [128]. Results are conveyed after a remote analysis of the signals. A similar plan of action can be executed for other diseases, including diabetes.

The fact that exhaled breath is an indicator of the physiological and metabolic processes of the human body consolidates the relevance of breath analysis for health and disease diagnostics, but it also points towards the associated hurdles. Most of the current findings are the results of open trials performed in a highly controlled environment. The effect of confounding variables, such as diet, exposure to a certain atmosphere, prior glycemic control, tissue complications, and physical activity need to be studied before commenting on the real-life applicability of any approach [10]. Diabetes monitoring through the exhaled breath analysis is indeed complex, because it is an indirect methodology that relies on monitoring the human body’s metabolic processes through the associated VOC biomarkers instead of direct blood sampling. Besides the recently explored biofluids, respiratory fluid can also assist in non-invasive glucose monitoring as it has rapid and stable glucose exchange with plasma. There happens to be an increase in glucose concentration of respiratory fluid in hyperglycemia. Exhaled breath condensate (EBC) glucose is estimated to have a dilution factor of 1:10,000 from plasma glucose [129]. Glucose sensing through EBC could be a breakthrough in non-invasive monitoring, but a few concerns, including glucose dilution, sample stability, and subject variability, need to be alleviated [129].

A crucial step in breath analysis is breath sampling. Exhaled breath broadly consists of mixed expiratory, late expiratory, and end-tidal phases.

- Mixed expiratory breath includes all the phases of breath and is prone to environmental, nose, and mouth contaminants.
- Removal of the estimated dead space from the breath results in the late expiratory breath. It has a better concentration of endogenous VOCs.
- End-tidal breath has the highest level of exhaled CO_2_ and is the richest in endogenous VOCs.

End-tidal breath is most preferred for breath analysis, but it does not encompass the possibility of all biomarkers as some of the gas exchanges happen in the upper airways. Therefore, the target breath biomarkers govern the choice of selecting the part of exhaled breath for sampling. For example, nitric oxide, often used for clinical characterization of asthma, originates from the airway. Hence, end-tidal breath only is inappropriate for its monitoring [130].

Depending upon the breath-sampling technique, VOCs may dilute, leading to hurdles in detection and sensing. Moisture can act as a cross-reactive agent, thus interfering with the analysis. Here, pre-concentration comes to the rescue, but it also adds delay to the real-time analysis. Thermal desorption tubes are most commonly used for pre-concentration [130]. The sorbent packed in the tube determines the range of volatiles that can be trapped, along with the stability and reproducibility. The solid-phase microextraction (SPME) technique uses a coated fiber for pre-concentration [130]. Since SPME follows an equilibrium-driven approach, environmental factors, such as temperature, particle loading, and sample flow rate influence its performance [131]. Needle trap is a solventless sampling method in which sorbents inside needle-like devices are used as an extraction trap [132]. This technique is known for its lower sampling time and volume, improved detection limits, stability, and reproducibility [133].

An exhaled breath flow rate also influences the sensor response, making direct analysis tougher [8]. A study on online breath analysis for lung cancer included a rotameter in the developed multisensory system to monitor and control the exhalation rate [134]. An offline analysis is way more common, but it has a risk of delayed diagnosis. Furthermore, the storage of breath for offline analysis is unreliable, owing to the possibility of degradation of the sample. The concentration of VOCs gets altered after some time of storage, and the VOC signature associated with the collection bags also disturbs the actual composition of the sample. Additionally, storing the samples at a specific temperature (37 °C) is essential [135]. As is evident from the discussion, various aspects govern the success of breath analysis.

## 6. Conclusions

The low compliance towards invasive blood-glucose monitoring is pervasive. Up to 60% of people with T1DM and 67% with T2DM do not implement the required glucose-monitoring schedule [5]. Inadequate blood-glucose monitoring leads to long-term health consequences of diabetes. The development of feasible monitoring technologies is instrumental for adherence to the self-monitoring of blood glucose. Invasive testing yields precise results. However, the risk of skin infections and the associated pain makes it unsuitable for continuous glucose monitoring. Research on non-invasive blood glucose monitoring has led to the development of various devices, easing diabetes management through comfortable, minimally invasive/non-invasive continuous blood-glucose monitoring. Nevertheless, they still suffer from issues such as lag time and the requirement of frequent calibration. Breath analysis is a budding domain for non-invasive disease diagnosis and monitoring. It is safe, painless, and allows repetitive sampling. However, the intricacies of breath analysis need to be well-studied to enable reliable, accurate, and reproducible monitoring. The foundation for this approach includes the correct identification of breath biomarkers and associated metabolic pathways. E-noses are emerging as a solution to the lack of identification of specific biomarkers, but their success depends on the diversity and size of the reference library database. The next stage is breath sampling, which is a major determiner of the performance of a breath analysis system. The targeted phase of the exhaled breath is a cardinal point of concern. A sensing unit with proper limits of detection, sensitivity, selectivity, size, stability, durability, response/recovery time, and cost is vital for reliable analysis. Furthermore, the unit needs to be customized to sustain the effects of exhaled breath variables, such as breath flow rate, humidity, and temperature. Metal oxide semiconductor sensors are being extensively developed for breath analysis. Other materials, such as CNTs, graphene, semiconductor chalcogenides, and conductive polymers are also being explored. Besides the sensing material, the transduction mechanism also plays a crucial role in the performance of a sensing system. Chemiresistive, electrochemical, optical, and mass-sensitive sensors are some of the common categories employed in biosensing. Depending on the characteristics of the chosen sensing technique, other components, like a pre-concentration stage or humidity traps, must be included. Most studies have been conducted using limited cohorts. Open-trial and controlled-environment-based research results are insufficient to establish the supremacy of any proposed idea for breath analysis. Blind trials and parallel observation of confounding factors are imperative. Additionally, the adoption of standardization in breath-sampling, including both environmental factors and the used methods, is necessary to ensure reproducibility and scale-up of research efforts nationally and internationally. Joint efforts by researchers in the field of material science, clinical research, biotechnology, electronics, and information technology are primarily indispensable for the success of breath analysis as a non-invasive healthcare approach.

## Figures and Tables

**Table 2:** Diseases with Breath Biomarkers Overlapping with Diabetes Breath Biomarkers.

Serial Number	Disease	Biomarkers Overlapping with Diabetes Breath Biomarkers	References
1.	Cystic Fibrosis	Ethanol, isopropanol, acetone, methanol	[59]
2.	Heart Failure	Acetone, ethanol	[60]
3.	Lung Cancer	Methanol, ethanol, acetone, isoprene, isopropanol, propane, undecane	[61]

**Table 4:** VOC Clusters for Diabetes Diagnosis.

Biomarker Clusters	Healthy/T1DM/T2DM Subjects	Method Used	Research Outcome	References
Acetone, methyl nitrate, ethanol, and ethylbenzene	17 healthy, 8 T1DM subjects	Gas Chromatography	Mean Correlation Coefficients All = 0.883 Healthy Subjects = 0.836 T1DM Subjects = 0.950	[82]
2-pentyl nitrate, propane, methanol, and acetone	17 healthy, 8 T1DM subjects	Gas Chromatography	Mean Correlation Coefficients All = 0.869 Healthy Subjects = 0.829 T1DM Subjects = 0.990	[82]
Acetone, ethanol, and propane	130 healthy, 70 subjects with diabetes	Analog Semiconductor Sensors	Mean Correlation Coefficients All = 0.25 Healthy subjects = 0.97 Subjects with diabetes = 0.35	[83]
Isopropanol, 2.3.4-trimethylhexane, 2,6,8-trimethyldecane, tridecane, and undecane	39 healthy, 48 T2DM subjects	Gas Chromatography—Mass Spectrometry	Sensitivity = 97.9% Specificity = 100%	[79]

**Table 5:** Recently Developed Acetone-Selective MOS Sensors.

Material	Operating Temperature	Detection Limit	Response Time/Recovery Time	References
Stable cobalt chromite (CoCr_2_O_4_)	300 °C	1 ppm	1.65 s/62 s (1 ppm)	[92]
Pt−Zn_2_SnO_4_ hollow octahedra	350 °C	Theoretical detection limit: 1.276 ppb for Pt10–ZTO sensor (Pt loading amount of 1 wt%)	14 s/607 s (100 ppm)	[93]
Cu-doped p-type ZnO nanostructures	Room Temperature	1 ppm	450 s/100 s	[94]
SnO_2_ nanosheet structure, with mainly exposed (101) crystal facets	280 °C	110 ppb	40 s/610 s (1 ppm)	[95]
WO_3_	300 °C	<1 ppm	24 s/27 s	[96]

## Data Availability

Not applicable. No new data were created or analyzed in this study.
